# Exchange of genetic information between therian X and Y chromosome gametologs in old evolutionary strata

**DOI:** 10.1002/ece3.3278

**Published:** 2017-09-12

**Authors:** Peter Peneder, Barbara Wallner, Claus Vogl

**Affiliations:** ^1^ Institut für Tierzucht und Genetik Veterinärmedizinische Universität Wien Vienna Austria; ^2^ Department für Mikrobiologie, Immunbiologie und Genetik Zentrum für Molekulare Biologie Universität Wien Vienna Austria

**Keywords:** comparative method, evolutionary strata, gene conversion, sex chromosome evolution, therians

## Abstract

Therian X and Y sex chromosomes arose from a pair of autosomes. Y chromosomes consist of a pseudoautosomal region that crosses over with the X chromosome and a male‐specific *Y*‐chromosomal region that does not. The X chromosome can be structured into “evolutionary strata”. Divergence of X‐chromosomal genes from their gametologs is similar within a stratum, but differs among strata, likely caused by a different onset of suppression of crossing over between gametologs. After stratum formation, exchange of information between gametologs has long been believed absent; however, recent studies have shown limited exchange, likely through gene conversion. Herein we investigate exchange of genetic information between gametologs in old strata that formed before the split of Laurasiatheria (cattle) from Euarchontoglires (primates and rodents) with a new phylogenetic approach. A prerequisite for our test is an overall preradiative topology, that is, all X‐chromosomal gametologs are more similar among themselves than to Y‐chromosomal sequences. Screening multiple sequence alignments of the coding sequences of genes from cattle, mice, and humans identified four genes, *DDX3X/Y*,*RBMX/Y*,*USP9X/Y*, and *UTX/Y*, exhibiting a preradiation topology. Applying our test, we detected exchange of genetic information between all four X and Y gametologs after stratum formation.

## 1 INTRODUCTION

Therian sex chromosomes originated from a pair of autosomes, of which the proto‐Y chromosome acquired the sex‐determining region Y (*SRY*) (reviewed in Graves, 2006). The *SRY* is the master switch for male‐specific development (Berta et al., 1990; Koopman, Gubbay, Vivian, Goodfellow, & Lovell‐Badge, 1991) and presumably is a mutated version of its X‐chromosomal homolog, or gametolog, *SOX3* (Foster & Graves, 1994; Südbeck, Schmitz, Baeuerle, & Scherer, 1996). As monotremes do not possess the *SRY* gene, the event dates to after monotremes diverged from therians, but prior to the divergence of marsupials and eutherians (Veyrunes et al., 2008; Wallis et al., 2007).

Thereafter, genes with male‐specific functions accumulated on the Y chromosome. For females, gain of expression of a male‐specific gene would be harmful, and for males, loss of expression (Rice, 1984). Thus, an individual should either inherit all male‐specific genes, and become male, or inherit none, and become female. Tight genetic linkage, either from the close proximity of two genes or from inhibition of recombination between them, is thus strongly selected for as soon as two male‐specific genes accumulate on a proto‐Y chromosome (Bull 1983; Fisher, 1931; Nei, 1969). This selective pressure has presumably led to the accumulation of male‐specific genes near the *SRY* and to the inhibition of meiotic recombination between them, resulting in a Y chromosome consisting of (1) a pseudo‐autosomal region (PAR), where crossover between the sex chromosomes is still possible and (2) a male‐specific Y‐chromosomal region (MSY) in which recombination is suppressed. The lack of recombination between parts of the X and Y chromosomes has resulted in the differentiation of allelic variants on the X and Y chromosomes, that is, the differentiation of gametologs (Garcia‐Moreno & Mindell, 2000); for a review, see, for example, Ellegren (2011).

Strikingly, the therian Y chromosome is degraded, such that it contains only few functional genes but many repetitive sequences, whereas gene content and repetitiveness of the X chromosome are similar to autosomes. Genes on the MSY are thought to degenerate (Graves, 2006) due to

1. a higher mutation rate: The microenvironment of the Y chromosome in the testes boosts mutations due to a lack of repair enzymes, oxidative stress, and a high number of cell divisions.
2. the inefficiency of selection on the Y chromosome due to lack of recombination: Recombination generates variance in fitness, which provides opportunity for selection, whereas genetically linked regions are inherited (and selected for) as a whole. Deleterious mutations then cause gradual degeneration of the linked genes on the Y chromosome (Muller's ratchet).

As a result, most genes on the therian Y chromosome have been lost completely during evolution, some have remained only as pseudogenes, that is, dysfunctional DNA sequences that are no longer expressed. Several preserved Y‐chromosomal genes with homologs on the X chromosome have acquired a male‐specific function. The high proportion of Y‐chromosomal genes with male‐specific function can be explained by strong selection preventing degeneration. Some genes on the Y chromosome have counterparts on the X chromosome that escape X chromosome inactivation in females (Lahn & Page, 1997). As these genes are thus expressed on both chromosomes in females, selective pressure on the Y‐chromosomal gene may act to retain the proper gene dosage also in males. Stabilizing selection on the male copy may then prevent degeneration (reviewed in Ellegren, 2011; Bachtrog, 2013). Additionally, other male advantage genes with no known X homolog appear to have been transposed to the Y chromosome from autosomes (reviewed in Graves, 2006).

### 1.1 Evolutionary strata

**Figure 1:**
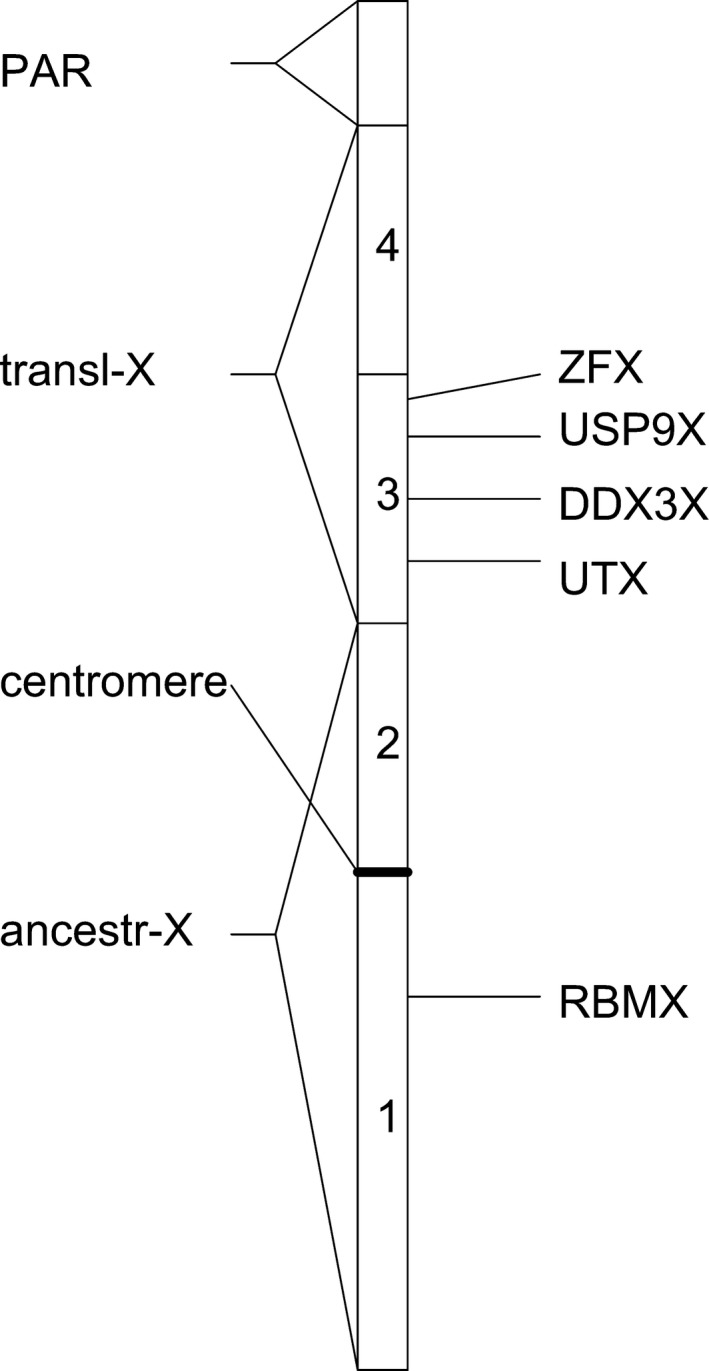
Schematic representation of the strata of a eutherian X chromosome. Numbers 1–4 represent the strata according to Lahn and Page (1999), “ancestr‐X” refers to the original X chromosome, “transl‐X” to the translocated region of eutherians, and “PAR” to the pseudo‐autosomal region. The genes considered in this article are indicated at their relative positions

Mechanisms inhibiting recombination likely involved chromosomal rearrangements (especially inversions) (Lahn & Page, 1999; Lemaitre et al., 2009) and occurred in successive evolutionary events, each suppressing recombination between X and Y chromosomes in a certain region, or “evolutionary stratum.” Gametologs in older strata show higher divergence. Four such evolutionary strata have been identified in human sex chromosomes by Lahn and Page (1999); later studies differentiate five (Ross et al., 2005) or even nine (Pandey, Wilson Sayres, & Azad, 2013). The position of the strata on the X chromosome corresponds to the timing of the stratum formation. The position of gametologs on the Y chromosome generally does not correspond to the strata on the X chromosome, which indicates that rearrangements on the Y chromosome might indeed have been responsible for the inhibition of recombination (Lahn & Page, 1999). Note, however, that Katsura and Satta (2012) shows that the pairwise divergence times of genes in strata one and two differ little between marsupials and eutherians. Graves and colleagues (Graves, 1995, 2006) showed that the third and fourth strata of Lahn and Page (1999), located on the short arm of the eutherian X chromosome, arose through a translocation from an autosome (see Figure 1). As this translocation is present in all eutherians but not in marsupials, it must have occurred in the ancestor of extant eutherian mammals after divergence from the metatherians, that is, between about 180 and 100 MYA. Because of this chromosomal rearrangement, the eutherian X and Y chromosomes should be considered neo‐X and neo‐Y chromosomes. Thus, the boundary between strata two and three also corresponds to the boundary of a chromosomal rearrangement.

### 1.2 Genetic exchange between the sex chromosomes

Presently, only the PAR exhibits crossovers between the X and Y chromosomes. In the PAR, gametologs are nearly identical, which is necessary for pairing and proper segregation of the sex chromosomes during meiosis (Burgoyne, 1982). The borders of the PAR, that is, the pseudo‐autosomal boundaries, sometimes seem to be displaced during evolution by attrition, but details remain unclear (Van Laere, Coppieters, & Georges, 2008). In contrast to the PAR, the MSY is generally highly differentiated from the X chromosome. However, limited genetic exchange is apparently still possible, most likely through gene conversion.

Gene conversion is the unidirectional transfer of genetic information from a donor to an acceptor sequence. It can occur between allelic sequences, but also between nonallelic sequences with greater than 90% sequence identity. These nonallelic sequences may be located on the same or on different chromosomes (reviewed in Chen, Cooper, Chuzhanova, Férec, & Patrinos, 2007). On sex chromosomes, gene conversion can commonly be observed between Y‐multicopy genes (i.e., genes with more than one Y‐chromosomal paralog), but, importantly, in few cases also between gametologs outside the PAR in recombination‐suppressed strata. Previous studies showed gene conversion between the gametologs of *ZFX/Y* (e.g., Pecon Slattery, Sanner‐Wachter, and O'Brien (2000)), *KALX/Y* (Iwase, Satta, Hirai, Hirai, & Takahata, 2010; Skaletsky et al., 2003), *VCY*,* ARSD*, and the *ARSDP* pseudogene (Iwase et al., 2010; Skaletsky et al., 2003; Trombetta, Cruciani, Underhill, Sellitto, & Scozzari, 2010; Trombetta, Sellitto, Scozzari, & Cruciani, 2014), *AMELX/Y*,* TBL1X/Y*,* STS*,* NLGN4*, and *PRKX/Y* (Pessia, 2013; Trombetta et al., 2014), and in the regions surrounding the latter gene (Rosser, Balaresque, & Jobling, 2009; Trombetta et al., 2014). All these genes are located in strata seven to nine according to Pandey et al. (2013). Gene conversion close to the transition between the PAR and the MSY may be explained by a leaky boundary. Katsura and Satta (2012) provide evidence for gene conversion in the eutherian lineage between *SMCX/Y* and *UBE1X/Y*, which are located in stratum six according to Pandey et al. (2013), with a test based on comparing regions within genes. Gene conversion may occur to repair double‐strand breaks, when only the gametolog is available as template to repair double‐strand breaks (Trombetta et al., 2014). As only little exchange of genetic information is necessary to prevent the rapid degeneration on the Y chromosome (Pecon Slattery et al., 2000), these gene conversion events challenge current models of the evolution of Y chromosomes presented above.

In this article, we investigate evolutionarily old strata, where crossover had already been suppressed since before the split of the three taxa we investigate: cattle (of the order Artiodactyla), mice (of the order Rodentia), and humans (of the order Primates). Aligned sequences of genes in these strata generally show site patterns consistent with a preradiation topology with respect to the split between, on the one hand, Laurasiatheria (including artiodactylans) and, on the other, Euarchontoglires (including primates and rodents) (see subsection 2.3 for details). This split dates to about 84 MYA and the split between Euarchonta and Glires to about 76 MYA (dos Reis et al., 2012). Note that a gene conversion within rodents or primates must have occurred at least 8 million years after stratum formation. Our aim is to detect evidence of gene conversion that occurred after the inhibition of crossover of these strata. We screened all available genes; however, only few functional genes have remained on the Y chromosome, specifically within rodents only a handful. Indeed, some rodents have entirely lost their Y chromosome (reviewed in Graves, 2006). Furthermore, sequencing and annotation of the Y chromosome is difficult, because of the largely repetitive Y‐chromosomal sequence, such that reliable Y‐chromosomal sequences are only available from few species.

We aligned MSY sequences, as frequencies of substitution site patterns of homologous sites may provide information about evolution. With a variant of the “co‐double method” (Balding, Nichols, & Hunt, 1992), we compare frequencies of substitution site patterns that (1) can only arise by double substitutions with those that (2) may arise by either a double substitution or a single substitution and subsequent gene conversion between gametologs. An excess of the latter indicates exchange of information between gametologs after stratum formation. A prerequisite for this test is an overall preradiative topology, that is, all X‐chromosomal sequences are more similar among themselves than to Y‐chromosomal sequences. Statistical power for our study mainly comes from the number of sites, rather than from the number of species studied. Adding more species would have reduced the number of sites or maybe even the number of genes further. We screened all genes in the MSY that could be aligned in these three well‐studied taxa and the outgroup platypus.

## 2 MATERIALS AND METHODS

### 2.1 Gene sequences and multiple sequence alignment

According to our screening of the databases, only five genes potentially satisfy our criteria: *RBMX/Y*,* DDX3X/Y*,* USP9X/Y*,* UTX/Y* and *ZFX/Y*. Coding sequences of these genes in humans (*Homo sapiens*), mice (*Mus musculus*), and cattle (*Bos taurus*) as well as the coding sequence of the respective homologs in platypus (*Ornithorhynchus anatinus*) were obtained from http://www.ncbi.nlm.nih.gov (Table 1). The locations of these genes in strata according to Lahn and Page (1999) and Pandey et al. (2013) are given in Table 2, along with information on X‐inactivation in humans and mice. Further information on location of the genes on the X and Y chromosomes, on paralogs, evolution, function, and expression is given in the Data S1.

**Table 1:** Accession numbers of sequence information used in this publication. Only the coding sequences were used. In the case of the murine *Zfy* gene, additionally to the *Zfy2* gene listed below, an alignment with *Zfy1* (NM_009570.4) was performed, which led to similar results

Gene	*B. taurus*	*H. sapiens*	*M. musculus*	*O. anatinus*
*DDX3X*	NM_001192962.1	NM_001356.4	NM_010028.3	XM_007667286.1
*DDX3Y*	NM_001172595.1	NM_001122665.2	NM_012008.2	
*RBMX*	NM_001172039.1	NM_002139.3	NM_011252.4	XM_007666005.1
*RBMY*	GU304599.2	XM_011531439.1	NM_011253.2	
*USP9X*	XM_010822079.1	XM_005272675.3	XM_011247454.1	XM_007667255.1
*USP9Y*	FJ627275.1	XM_011531469.1	NM_148943.2	
*UTX*	NM_001206575.1	NM_001291415.1	NM_009483.2	XM_007667229.1
*UTY*	XM_010802496.1	NM_001258267.1	NM_009484.2	
*ZFX*	NM_177490.1	NM_003410.3	NM_001044386.1	XM_001515746.2
*ZFY*	NM_177491.1	NM_003411.3	NM_009571.2	

**Table 2:** Characteristics of genes: stratum according to Lahn and Page (1999) (LP99) and Pandey et al. (2013) (PAND13); preradiation (PRR) versus postradiation phylogeny; X‐inactivation in humans (XIH) and mice (XIM); and the copy number of the gene in the whole genome (see also the Data S1)

Gene	LP99	PAND13	PRR	XIH	XIM	Copies
*DDX3X/Y*	3	6	Yes	No	No	Some
*RBMX/Y*	1	2	Yes	Yes	Yes	Many
*USP9X/Y*	3	6	Yes	No	No	Many
*UTX/Y*	3	6	Yes	No	No	Few
*ZFX/Y*	3	7	No	No	Yes	Few

For all five genes, multiple sequence alignments (MSAs) of all seven sequences were produced using T‐coffee (Notredame, Higgins, & Heringa, 2000). The outgroup species platypus was used for polarization of the states. For further investigation, we only used sites on which the MSA consists of exactly two different variants, because these are likely not hypermutable, as is plausible for sites which include three different nucleotides.

### 2.2 Binary site patterns

Frequencies of substitution site patterns in a multiple sequence alignment can provide information about evolution. Assuming no gene conversion and the parsimony principle, evolutionary scenarios with the fewest evolutionary changes most likely explain the observed data. In our context, this favors the model that explains the observed site pattern with the least number of substitutions.

At a given analyzed site, we code every possible combination of the nucleotide variants of all species in a binary number of six digits. The platypus (*Ornithorhynchus anatinus*) nucleotide variant is set to 0 and the alternative variant to 1. The first digit corresponds to the nucleotide variant of the *Bos taurus* X chromosome at that particular site (henceforth abbreviated as *bx*), the second corresponds to the *Bos taurus* nucleotide variant of the Y chromosome (*by*), the third and fourth digits correspond to the nucleotide variants of the *Homo sapiens* X and Y chromosomes (*hx* and *hy*, respectively), and the fifth and sixth digits correspond to the *Mus musculus* X‐ and Y‐chromosomal nucleotide variants (*mx* and *my*, respectively). Note that an additional substitution on the branch connecting the three species to platypus only exchanges the 1′s and 0′s; we treat such inverse site patterns equivalently.

A binary site pattern of, for example, 10 10 10 suggests a preradiation topology and a single substitution on the X gametolog after the split from the Y gametologs, but before the split of Laurasiatheria (cattle) from Euarchontoglires (primates and rodents). A binary site pattern of, for example, 00 10 10 suggests a single substitution on the X gametolog on the stem connecting humans and mice, that is, after the X gametologs split from the Y gametologs and after the phylogenetic split of cattle from the other two species, but before the split of primates from rodents. We code such a site pattern, together with a substitution on the cattle branch, for example, the site pattern 10 00 00, as *b*ovine and *s*tem substitutions on the X chromosome, that is, as *bsx*, and the corresponding substitution site pattern on the Y chromosome as *bsy* (see Table 3).

**Table 3:** Substitution site patterns and the corresponding double substitutions

Within‐lineage		Among‐lineage	
Site pattern	Substitutions	Site patterns	Substitutions
00 00 11	*mx *+ *my*	10 01 00, 01 10 00	*bsx *+ *hy*,* bsy *+ *hx*
00 11 00	*hx *+ *hy*	01 00 10, 10 00 01	*bsy *+ *mx*,* bsx *+ *my*
11 00 00	*bsx *+ *bsy*	00 10 01, 00 01 10	*hx *+ *my*,* hy *+ *mx*
00 11 11	*bsx *+ *bsy*	11 10 01, 11 01 10	*hy *+ *mx*,* hx *+ *my*
11 00 11	*hx *+ *hy*	10 11 01, 01 11 10	*bsy *+ *mx*,* bsx *+ *my*
11 11 00	*mx *+ *my*	10 01 11, 01 10 11	*bsy *+ *hx*,* bsx *+ *hy*

The linkage structure may also provide information about underlying processes. We thus looked for autocorrelation among site patterns along the alignment. In particular, we compared site patterns that suggest a gene conversion along the rodent branch, that is, 00 00 11, to the theoretical expectation if there is no autocorrelation, that is, the geometric distribution, using Q‐Q plots (R Core Team, 2015). We chose the rodent branch, because it is the only branch with enough substitutions that makes detection of deviation likely. As we observed little autocorrelation, we treated each site of the MSA independently. Similarly, function may constrain evolution. We therefore compared the synonymous/nonsynonymous and transition/transversion rates between different groups of patterns for all genes, using chi‐square tests.

### 2.3 Test for gene conversion

**Figure 2:**
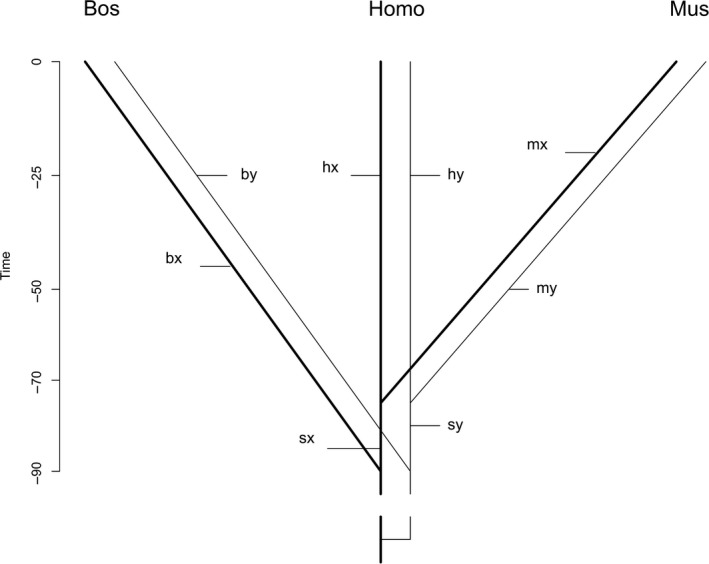
Preradiation phylogeny of X and Y gametologs in the three species *Bos*,* Homo*, and *Mus*. Time is measured in millions of years before present. The X gametolog is characterized by thick lines and the Y gametolog by thin lines. The broken lines at the base indicate a variable time of differentiation of gametologs before the split of the Laurasiatheria from the Euarchontoglires. *bx*,* hx*,* mx* are the terminal branches of the *X* gametolog phylogeny leading to cattle, humans, and mice, respectively, and *sx* is the internal branch uniting the Euarchontoglires humans and mice after the split of Laurasiatheria. *by*,* hy*,* my*, and *sy* are the respective branches on the *Y* gametolog

For the co‐double method, the divergence between X and Y gametologs must precede the earliest relevant phylogenetic split between Laurasiatheria (cattle), on the one hand, and Euarchontoglires (humans and mice), on the other (Figure 2). The orthologs on the X chromosome should thus be more closely related to each other than to their respective gametologs on the Y chromosome, that is, the gene topology should be consistent with a “preradiation topology” (Wilson & Makova, 2009). For each gene, we constructed a phylogenetic tree with the neighbor‐joining method (Saitou & Nei, 1987) and evaluated consistency with a preradiation topology using the binary site patterns and a parsimony principle.

The proposed test compares the frequencies of substitution site patterns that (1) can only arise by double substitutions with those that (2) may arise by either a double substitution or a single substitution and subsequent gene conversion between gametologs. The reasoning is as follows: Given a preradiation topology and in the absence of gene conversion, for some site patterns double substitutions need to be assumed, for example, for 00 11 00 or 01 10 11. The first site pattern 00 11 00 can also be produced by a single substitution of either the X‐ or the Y‐chromosomal nucleotide of humans and a gene conversion within the human lineage. In contrast, the second site pattern can only be produced by double substitutions, because after the split of the Laurasiatheria from the Euarchontoglires, the X‐chromosomal gametolog of cattle and the Y‐chromosomal gametolog of humans were never in the same organism.

The substitution site patterns of the gametologs with the most parsimonious double substitutions are summarized in Table 3. The (double) substitutions producing the site patterns on the left side occur within a lineage, whereas those on the right side occur among different lineages. Note that summing all site patterns and substitutions to the left in Table 3, that is, the within‐lineage site patterns, results in 2·(*bsx* + *bsy* + *hx* + *hy* + *mx* + *my*); summing all site patterns to the right, that is, the among‐lineage site patterns, results in 4·(*bsx* + *bsy* + *hx* + *hy* + *mx* + *my*). Therefore, we expect a ratio of within‐lineage site patterns (to the left) to among‐lineage site patterns (to the right) of 1:2, as a double substitution is twice as likely to cause an among‐lineage site pattern than a within‐lineage site pattern. Note that, even if further mutations became fixed or if mutation rates differed among species, a ratio of 1:2 would be expected, unless the mutation bias among the four nucleotides would differ systematically among species. The within‐lineage site patterns on the left side of Table 3 could also be produced by a single substitution and subsequent gene conversion. This would lead to a ratio greater than 1:2. We can thus test the observed frequency of within‐ to among‐lineage site patterns with a one‐sided test. If the observed proportion significantly exceeds 1:2, gene conversion is likely to have contributed in addition to double substitutions. Note that other binary site patterns or sites with apparent single or triple substitutions may also contain information about the rate of double substitutions and gene conversions, but cannot easily be interpreted and are thus omitted. In our implementation, we conditioned on the total number *N* of binary site patterns given by the data for each gene. We then drew repeatedly from a binomial distribution with “probability” *p *=* *1/3 and with the “sample size” *N* set the total number of double substitutions of all site patterns per gene (Table 3). For a one‐sided test, we set the *p*‐values to the proportion of draws at least as extreme as the observed frequencies.

## 3 RESULTS

**Figure 3:**
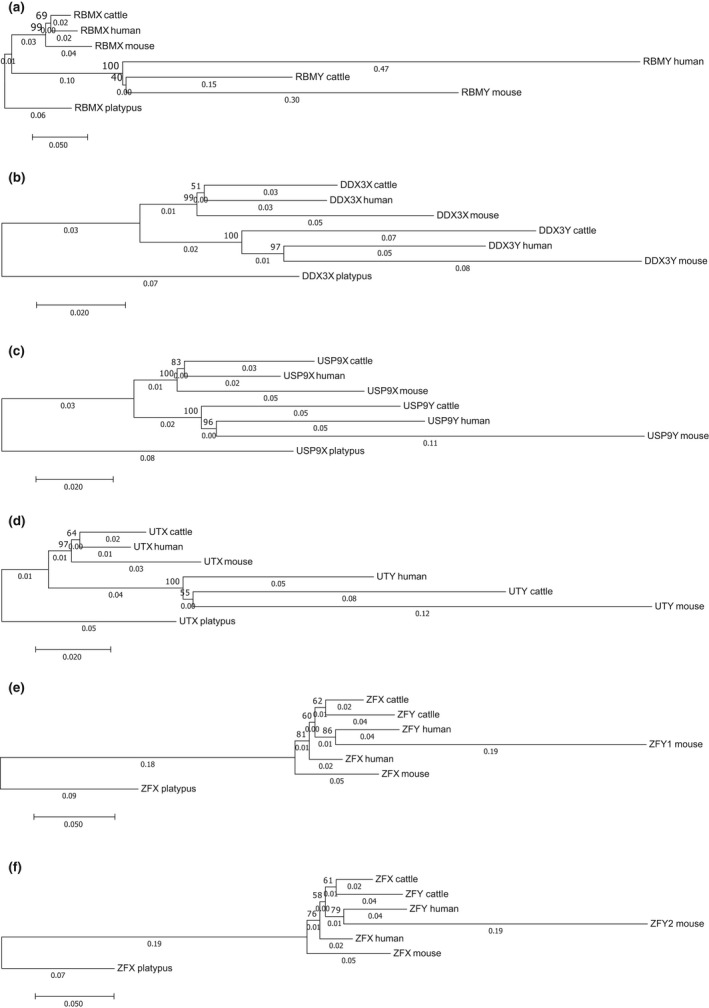
Neighbor‐joining trees of the genes (a) *RBMX/Y*, (b) *DDX3X/Y*, (c) *USP9X/Y*, (d) *UTX/Y*, and *ZFX/Y* with the two mouse paralogs (e) *Zfy1* and (f) *Zfy2*. The percentage of replicate trees in which the associated taxa clustered together in the bootstrap test (1,000 replicates) is shown next to the nodes. The trees are drawn to scale, and the branch lengths (shown next to the branches) represent the number of base substitutions per site. The evolutionary distances were computed using the maximum composite likelihood method (Tamura, Nei, & Kumar, 2004). All positions containing gaps were excluded from analysis. Evolutionary analyses were conducted in MEGA7 (Kumar, Stecher, & Tamura, 2016)

The gene pairs *DDX3X/Y*,* RBMX/Y*,* USP9X/Y*, and *UTX/Y* from cattle, mice, and humans showed a preradiation topology, when phylogenies were inferred using neighbor joining (Figure 3). The gametologs *ZFX/Y* showed an unclear topology and were excluded from further studies. Note that in most cases, the branches of Y gametologs are longer, which reflects the higher mutation rates on Y chromosomes. The longer branch lengths of mice compared to the other species are most likely a result of the shorter generation time of rodents. All topologies were confirmed by examining the substitution site patterns applying the parsimony principle (results not shown).

**Figure 4:**
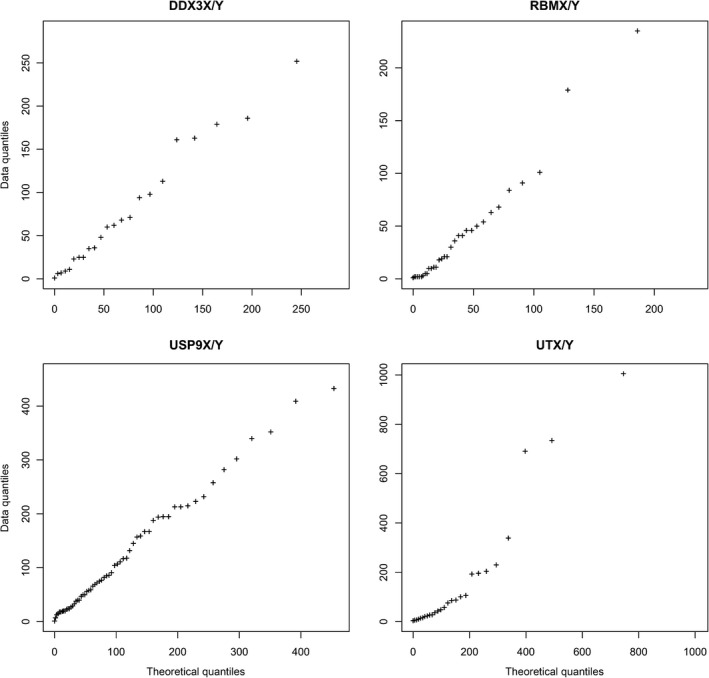
Q–Q plots of distances between neighboring site patterns. The empirical data for the genes DDX3, RBM, USP, and UTX are compared to the geometric distribution, which assumes absence of autocorrelation

The lengths of the MSAs without gaps of the four gene pairs *DDX3X/Y*,* RBMX/Y*,* USP9X/Y*, and *UTX/Y* are given in Table 4. The high frequency of variant site patterns in mice allowed comparison of spatial patterns with the expectation of no spatial aggregation, that is, a geometric distribution. In particular, within‐lineage site patterns supportive of gene conversion were most frequent within the rodent branch (DDX3: 24 of 30; RBM: 24 of 42; USP9: 61 of 96; and UTX: 27 of 33). Deviation of the distance of consecutive site patterns within the rodent lineage from the geometric expectation was generally weak as shown by Q–Q plots (Figure 4); only in the case of UTX/Y, deviation from the theoretical expectation was evident. Because of this negligible autocorrelation, inference of gene conversion tracts was not attempted.

**Table 4:** Gene names, lengths of the multiple sequence alignments in number of sites without gaps, absolute numbers of within‐lineage and among‐lineage site patterns, and *p*‐values of the one‐sided test of the expectation of only double substitutions

Gene	Gene length	Within‐lineage	Among‐lineage	*p*‐value
*DDX3X/DDX3Y*	1,901	30	35	.0199
*RBMX/RBMY*	950	35	28	.0003
*USP9X/USP9Y*	7,490	96	113	.0002
*UTX/UTY*	2,818	33	42	.0390

We note that no differences between transitions and transversions in within‐ and among‐lineage site patterns could be observed (Table 5). Likewise, proportions of within‐ and among‐lineage patterns between synonymous and nonsynonymous substitutions were similar, but numbers of nonsynonymous substitutions were too low for meaningful tests (Table 6).

**Table 5:** Within‐ and among‐lineage site patterns of the four genes *DDX3X/Y*,* RBMX/Y*,* USP9X/Y*, and *UTX/Y* analyzed for transitions and transversions. Significance testing was performed with a chi‐square test; *p*‐values were determined from simulated data

Gene	Transition within‐lineage	Transition among‐lineage	Transversion within‐lineage	Transversion among‐lineage	*p*‐value
*DDX3X/DDX3Y*	27	30	3	5	.73
*RBMX/RBMY*	35	28	7	10	.39
*USP9X/USP9Y*	85	116	11	14	1
*UTX/UTY*	28	38	5	4	.52

**Table 6:** Codons that include within‐ and among‐lineage site patterns of the four genes *DDX3X/Y*,* RBMX/Y*,* USP9X/Y*, and *UTX/Y* analyzed for synonymous and nonsynonymous substitutions. Only codons for which the MSA consists of exactly two different variants were used. In order to increase the number of these codons, platypus was excluded from analysis

Gene	Synonymous within‐lineage	Synonymous among‐lineage	Nonsynonymous within‐lineage	Nonsynonymous among‐lineage
*DDX3X/DDX3Y*	1	2	16	17
*RBMX/RBMY*	0	0	1	0
*USP9X/USP9Y*	6	9	34	54
*UTX/UTY*	1	0	4	4

We thus lumped all data for the analyses with the variant co‐double method. The frequencies of within‐ and among‐lineage substitution site patterns were compared with a one‐sided test to the proportion of 1:2, which is expected assuming only double substitutions (Table 4). For all four genes, the expected proportion was rejected (*p *<* *.05). (Note that a two‐sided chi‐square test for *UTX/Y* would be just nonsignificant with *p *=* *.05004.) We thus accepted the alternative hypothesis that information was exchanged between the X and Y gametologs, that is, that gene conversion contributed to the observed site patterns. The frequencies of the site patterns differ among genes (Table 7).

**Table 7:** Substitution site patterns, the corresponding necessary double substitutions or gene conversions (g.c.), and their counts for each of the four genes *DDX3X/Y*,* RBMX/Y*,* USP9X/Y*, and *UTX/Y*

Site pattern	Substitutions/g.c.	*DDX3X/Y*	*RBMX/Y*	*USP9X/Y*	*UTX/Y*
00 00 11	*mx *+ *my*	17	29	43	23
00 11 00	*hx *+ *hy*	3	3	9	2
11 00 00	*bsx *+ *bsy*	1	0	9	0
00 11 11	*bsx *+ *bsy*	3	1	12	5
11 00 11	*hx *+ *hy*	0	0	6	0
11 11 00	*mx *+ *my*	6	2	17	3
00 01 10	*hy *+ *mx*	8	21	24	11
00 10 01	*hx *+ *my*	2	0	20	6
01 00 10	*bsy *+ *mx*	2	1	8	1
01 10 00	*bsy *+ *hx*	5	1	16	5
01 10 11	*bsx *+ *hy*	2	0	5	1
01 11 10	*bsx *+ *my*	1	0	1	1
10 00 01	*bsx *+ *my*	2	1	11	2
10 01 00	*bsx *+ *hy*	2	0	12	4
10 01 11	*bsy *+ *hx*	3	0	6	4
10 11 01	*bsy *+ *mx*	1	0	0	1
11 01 10	*hx *+ *my*	4	0	10	0
11 10 01	*hy *+ *mx*	3	4	17	6

## 4 DISCUSSION

Using a comparative test, we detect evidence for exchange of information between X and Y gametologs after stratum formation, likely due to gene conversion. For the test, we use a phylogenetic method and compare substitution site patterns in four pairs of gametologs (*DDX3X/Y*,* RBMX/Y*,* USP9X/Y*, and *UTX/Y*) in cattle, mice, and humans. In particular, we contrast frequencies of substitution site patterns that (1) can only arise by double substitutions with those that (2) may arise by either a double substitution or a single substitution and subsequent gene conversion between gametologs. For the method to be applicable, the stratum containing the gametologs must have formed before the split of Laurasiatheria (cattle) from Euarchontoglires (rodents and primates), that is, must follow a preradiation topology.

The X and Y gametologs of *DDX3X/Y*,* RBMX/Y*,* USP9X/Y*, and *UTX/Y* show a preradiation topology, confirming the results of a previous study (Wilson & Makova, 2009). *ZFX/Y* was the only pair of analyzed gametologs that did not show a preradiation topology. Possible explanations are that (1) either *ZFX* and *ZFY* diverged after the split of Laurasiatheria and Euarchontoglires or (2) gene conversion events between gametologs homogenized large gene tracts, interfering with the detection of an overall preradiation site pattern. The latter explanation is consistent with the results of other studies, which detected gene conversion between gametologs on the 3′ end of the gene (for details on *ZFX/Y*, see the Data S1). We excluded *ZFX/Y* from further study.

With our test, we show for all analyzed genes with a preradiation topology (namely *DDX3X/Y*,* RBMX/Y*,* USP9X/Y*, and *UTX/Y*) that exchange of genetic information must still have occurred after the split of the gametologs. Gene conversion must have taken place after the stratum had formed and recombination thus had been suppressed. We interpret this as evidence that stratum formation does not fully prevent gene conversion,

More than half of the within‐lineage site patterns supportive of gene conversion occurred within the rodent branch. Note that the corresponding gene conversion events must have occurred after the split of rodents from primates, while stratum formation and thus differentiation of gametologs must have occurred before the split of Laurasiatheria (artiodactylans) from Euarchontoglires (rodents and primates). The concentration of detected gene conversion events is likely due to the shorter generation time in rodents, which increases substitution rates and thus may facilitate detection of gene conversion, but may also increase the gene conversion rate directly. The high frequency of site patterns consistent with double mutation or single mutation and gene conversion in rodents allowed comparison of spatial patterns with the expectation of no spatial aggregation, that is, a geometric distribution. Generally, deviation from expectation was weak in one gene (UTX) and absent in the others (Figure 4). Many of the gene conversion events might have occurred many million years ago and likely have been masked other processes.

Of the four genes investigated herein, Wyckoff, Li, and Wu (2002) already noticed gene conversion between gametologs of *RBMX/Y*, located in the old stratum 2 (Pandey et al., 2013), but do not present evidence for it. Previous studies (see the Section 1) showed gene conversion between gametologs in younger strata, seven to nine according to Pandey et al. 2013). Katsura and Satta (2012) provide evidence for gene conversion in the eutherian lineage between *SMCX/Y* and *UBE1X/Y*, located in the old stratum six (Pandey et al., 2013). We note that exchange of genetic information without recombination has also been observed in other contexts, for example, in allopolyploid plants with disomic inheritance (Ma, Li, Vogl, Ehrendorfer, & Guo, 2009). It thus seems to be more frequent than commonly thought.

We do not infer rates of gene conversion and mutations, which would be a harder problem. We also do not include information on the positions of potential gene conversion sites, which has been used to test for gene conversion (Sawyer, 1989), because little autocorrelation along alignments was evident, such that inference of tract lengths seemed impossible. Our test would not work if gene conversion rates were so high that the majority of sites were affected, because then the overall tree topology would no longer be preradiative (this might have been the case with *ZFX/Y*). With our approach, it is impossible to infer the original mutation, and thus impossible to infer the direction of gene conversion, that is, either from the X to the Y chromosome or the reverse. Furthermore, the power of a test is limited by the sample size, which corresponds to the number of sites per gene in our test. Note that even the shortest gene *RBMX/Y* showed evidence for gene conversion. The test is affected little by differences in substitution rates among sites or among species and, generally, provides a simple, fast, and robust way to detect exchange of genetic information between gametologs.

Only the four genes investigated herein and *ZFX/Y* could be aligned among our four study species platypus, mice, humans, and cattle. This is mainly because rodents, in our case mice, have very reduced Y chromosomes. Note that the power of the tests comes mainly from the number of aligned sites. Adding more species would likely have reduced this number and thus likely have reduced power. Excluding mice but adding Y‐chromosomal sequences from other mammalian orders, could possibly have increased the number of genes with multispecies alignments that could be tested with our method. At present, however, nearly complete coding sequences of Y chromosomes are rare.

## CONFLICT OF INTEREST

None declared.
