# Supplementary material for: Exchange of genetic information between therian X and Y chromosome gametologs in old evolutionary strata
Source: Ecol Evol. 2017 Sep 12;7(20):8478–87. doi: 10.1002/ece3.3278 (PMC5648654; doi:10.1002/ece3.3278)
Supplement: Supplementary file 1 [file ECE3-7-8478-s001.pdf]

1 Supplementary Section: Genes

1.1 *RBMX/Y*

The gametologs *RBMX* (RNA Binding Motif Protein, X-Linked; formerly known as *HN-RNP G*, heterogeneous nuclear ribonucleoprotein G) and *RBMY* encode RNA-binding proteins with various functions, including splicing (Lingenfelter et al., 2001).

Location, Paralogs and Evolution
Earlier studies place the human *RBMX* in stratum one (Lahn and Page, 1999; Ross et al., 2005); more recently, (Pandey et al., 2013) place it in the second of a total of nine strata. *RBMY* has been shown to be located on the Y chromosome since before the divergence of eutherian and metatherian mammals more than 130 million years ago (Mazeyrat et al., 1999). *RBMY* and *RBMX* probably evolved from a gene on the mammalian proto-X and -Y pair at least 130 million years ago (Delbridge et al., 1999; Bhowmick et al., 2007). Subsequently *RBMY* probably underwent a series of duplication events, both before and during primate evolution. The *RBMY* gene family now consists of about 30 genes and pseudogenes, located on both arms of the Y-chromosome. (Chai et al., 1998; Bhowmick et al., 2007). Wyckoff et al. (2002) found evidence for X-Y gene conversion in *RBMX/Y*, but do not provide details. Additionally to *RBMX* and the *RBMY* gene family, multiple processed copies of the genes (RBMX-like sequences: RBMXLs) have been identified on the human chromosomes 1, 4, 6, 9, 11, 20 and X. These RBMXLs are apparently retroposons of the original gene, considering their lack of introns (Lingenfelter et al., 2001). Sequence conservation in primates and great apes indicates that they arose long before humans emerged. Inversions, deletions and premature stop codons in some RBMXLs indicate pseudogenes; others seem to be intact. Two functional RBMXLs were found to be expressed in a tissue-specific manner. One is specifically expressed in the testis, possibly compensating *RBMX*, which is inactivated in male germ cells (Lingenfelter et al., 2001; Elliott et al., 2000).

Function and Expression
Human *RBMX* is subject to X-inactivation, ubiquitously expressed in almost all organs, and reportedly plays an important role as a regulator of cohesin (Matsunaga et al., 2012; Lingenfelter et al., 2001). It is necessary for normal brain development in zebrafish (Tsend-Ayush et al., 2005), and is also inactivated in mice (Yang et al., 2010). It has been shown that RBMX interacts with other splicing regulatory proteins and regulates alternative splice site selection in a concentration dependent manner as a part of the supraspliceosome (Heinrich et al., 2009). RBMY is also involved in the regulation of splicing, but it regulates different RNAs than RBMX. These differences in alternative splicing might contribute to sex-specific differences in mammals (Heinrich et al., 2009). Human *RBMY* is exclusively expressed in germ cells (Elliott et al., 1997), whereas murine *Rbmy* is also expressed in the nervous and visual system (McKee et al., 2005). Human *RBMY* likely plays a role in

spermatogenesis (Abid et al., 2013) and is also a candidate oncogene for male liver cancer (Tsuei et al., 2004).

1.2 *DDX3X/Y*

DDX3 (DEAD (Asp-Glu-Ala-Asp) box helicase 3), also known as *DBX/DBY*, is a subfamily of genes which includes two gametologs in theria: *DDX3X* on the X-chromosome (Park et al., 1998), and *DDX3Y* on the Y-chromosome (Sekiguchi et al., 2004).

Location, Paralogs and Evolution:
Human *DDX3X* is located on stratum three of four according to Lahn and Page (1999) and three of five according to Ross et al. (2005); Pandey et al. (2013) place *DDX3X* in the sixth of a total of nine strata. In humans, there are two additional, potentially pseudogenized paralogs: the copy on chromosome 4 probably originated from retroposition of *DDX3X*; the other on the X-chromosome likely arose by transposition of *DDX3Y* (Kim et al., 2001; Chang and Liu, 2010). In mice, besides *Ddx3x* and *Ddx3y*, two autosomal paralogs on chromosome 1 have been identified. Both likely originated from *Ddx3x* via retroposition (Chang and Liu, 2010; Mazeyrat et al., 1998), and one of it, the intronless gene *Pl10* (also known as *D1Pas1*), has been shown to be functional in mice and likely plays a role in spermatogenesis (Vong et al., 2006). *PL10* can also be found on the chromosome 15 in cattle. Bovine *PL10* has been proved to be active at the transcriptional level, but it remains unclear if it gives rise to a functional protein. (Liu et al., 2009; Chang and Liu, 2010). In the non-eutherian lineages, *PL10* is the only member of the *DDX3* subfamily. Notably, it has been suggested that there was positive selective pressure on *PL10/DDX3X/DDX3Y* during the course of therian evolution (Chang and Liu, 2010). Reportedly, on the marmoset Y chromosome, *DDX3Y* is present as a multi-copy gene family and signs of Y-Y gene conversion have been found there (Bellott et al., 2014), but to the best of our knowledge, gene conversion between *DDX3X* and *DDX3Y* has not been reported yet.

Function and Expression:
DDX3X is a multifunctional ATP-dependent RNA helicase (Ariumi, 2014). Its ATPase activity can be stimulated by numerous DNA-sequences and RNAs (Franca et al., 2007). DDX3X is involved in many different cellular processes, including translation (Lee et al., 2008), cell cycle regulation (Lai et al., 2010), apoptosis (Sun et al., 2008) and tumor development (Chao et al., 2006; Botlagunta et al., 2008). Moreover, it was shown that DDX3X interacts with viruses such as Hepatitis C and HIV and serves as one of their major targets (Owsianka, 1999; Yedavalli et al., 2004). Recently, it has also been reported that DDX3X plays a role in anti-viral immune response (IFN-type-1 pathway) (Gale and Foy, 2005; Saito and Gale, 2007). *DDX3X* was shown to escape X-inactivation in mice and humans (Yang et al., 2010; Lahn and Page, 1997). *DDX3Y* is a major factor in spermatogenesis in humans; its loss leads to azoospermia or oligozoospermia (Sekiguchi

et al., 2004). It is located in the AZFa (Azoospermia Factor a) interval of the MSY (Lahn and Page, 1997; Vogt et al., 1996). Both human genes, *DDX3Y* and *DDX3X*, are transcribed in various tissues; the protein DDX3Y, however, has only been found in male germ cells, whereas the protein DDX3X has been detected in numerous tissues (Ditton et al., 2004). New studies indicate distinct temporal protein expression patterns of both gametologs, especially in spermatogenesis (Ditton et al., 2004). A rescue experiment with temperature-sensitive hamster-cell lines by (Sekiguchi et al., 2004) further suggests that up to a certain point, human *DDX3Y* seems to be able to complement the immune function of a *DDX3X* knock-out. The same was true for murine *Ddx3y* and *Pl-10*. Conversely, however, *DDX3X* cannot rescue the loss-of-function of *DDX3Y* in human (Rosner and Rinkevich, 2007). Importantly, the functionality of murine and human *DDX3Y* is not equivalent, as the functional paralog *Pl10* takes over some of the functions of human *DDX3Y* in mice. Murine *Ddx3x* and *Ddx3y* are both ubiquitously expressed in various tissues, but *Ddx3x* is expressed at a much higher level (Vong et al., 2006). During X- and Y-chromosomal silencing during meiosis, transcription of both *Ddx3x* and *Ddx3y* decreases significantly, whereas *Pl10*-expression increases, apparently to compensate (Rosner and Rinkevich, 2007; Session et al., 2001).

1.3 *USP9X/Y*

USP9X (Ubiquitin Specific Peptidase 9, X linked; also known as *FAM*) and *USP9Y* (Ubiquitin Specific Peptidase 9, Y linked) are two gametologs that encode substrate specific ubiquitin peptidases (Hall et al., 2003; Lee et al., 2003). These proteins play important roles as a downstream regulators of ubiquitination processes and prevent the degradation of proteins.

Location, Paralogs and Evolution:
Apart from *USP9X* and *USP9Y*, over 30 pseudogenes exist on the human Y chromosome (Karro et al., 2007). *USP9X* is located close to *DDX3X*, on stratum three according to the classifications by Lahn and Page (1999) and Ross et al. (2005), or on stratum six according to (Pandey et al., 2013). It is highly conserved across vertebrates (Khut et al., 2007). The expression of human *USP9X* can rescue neuronal defects derived from *Usp9x* conditionally deleted mice (Homan et al., 2014).

Function and Expression:
USP9X has been shown to act in such diverse pathways as cell death, self renewal, oncogenesis, cell adhesion, tumor-suppression, neurodegeneration, pluripotency, developmental signaling and neural development (reviewed in Murtaza et al., 2015). It is expressed in various tissues, most notably in the brain, and escapes X-inactivation (Jones et al., 1996). In mice, USP9X is highly important for brain development (Stegeman et al., 2013), and in humans, it is a candidate gene for X-linked intellectual disability (Tarpey et al., 2009).

USP9X and *USP9Y* have a highly similar DNA sequence and may therefore have similar functions. However, in human brain tissue, a differential regulation of *USP9X* and *USP9Y* could be shown, suggesting that the functional overlap is incomplete (Xu et al., 2002). *USP9Y* is part of the AZFa (Azoospermia factor a) region and plays a role in spermatogenesis. However, it remains unclear whether this function is essential (Luddi et al., 2009; Krausz et al., 2006). In humans, *USP9Y* is expressed in various adult and embryonic tissues, whereas in mice *Usp9y* is expressed specifically in the testis (Brown et al., 1998).

1.4 *UTX/Y*

UTX (Ubiquitously transcribed tetratricopeptide repeat, X linked; also known as *KDM6A*, (lysine (K)-specific demethylase 6A) and *UTY* (also known as *KDM6C*) are a pair of gametologs located on the X- and Y-chromosome, respectively.

Location, Paralogs and Evolution:
UTX is located on stratum three according to earlier classifications (Lahn and Page, 1999; Ross et al., 2005); (Pandey et al., 2013) place *UTX* in the sixth of their nine strata. *UTY* was found to be under positive selection pressure (Gerrard and Filatov, 2005). NCBI lists one human *UTY*-like pseudogene on chromosome 21. To the best of our knowledge, there are no specific studies concerning the evolution of *UTX/UTY*, and no gene conversion events have been detected between *UTX* and *UTY* so far.

Function and Expression:
UTX is a histone 3 lysine 27 (H3K27) demethylase, and thus plays an important role in the regulation of gene expression, including Hox-gene regulation, the re-establishment of pluripotency and germ cell development in mice and humans (Mansour et al., 2012; Hong et al., 2007; Shahhoseini et al., 2013; Agger et al., 2007). UTX is also known to play a role in hematopoiesis in humans (Liu et al., 2012), and, notably, escapes X-inactivation in mice and humans (Greenfield et al., 1998). This presumably explains the heightened expression of *Utx* found in female murine brain and liver tissue in comparison to male tissue (Xu et al., 2008). *UTX* mutations can lead to histone H3 lysine methylation deregulation, which can cause cancer as well as the rare Kabuki-syndrome in humans (van Haaften et al., 2009; Banka et al., 2015). Independent from its catalytic activity as a histone-demethylase, UTX has also been shown to have other important functions, e.g. for the establishment of ectoderm and mesoderm in embryonic stem cells (Morales Torres et al., 2013; Wang et al., 2012). UTX mediates interactions between lineage-defining transcription factors and SWI/SNF remodeling complexes in mice (Miller et al., 2010). Furthermore, UTX has been found in a complex which mediates H3K4 methylation (Cho et al., 2007).

Human *UTY* is part of the AZFa region, possibly involved in spermatogenesis, and expressed in various tissues (Foresta et al., 2000). It contributes to graft/host interac-

tions following sex-mismatched organ and bone marrow transplantation (Warren et al., 2000; Greenfield et al., 1996) and was generally found to be alternatively spliced (Laaser et al., 2011). Murine and human UTY were claimed to lack H3K27 demethylase activity (Shpargel et al., 2012), but human UTY catalyzes demethylation of H3K27 peptides in vitro, analogously to UTX, but with reduced activity due to point substitutions (Walport et al., 2014). Previous claims that the function of UTY in development is independent of H3K27 demethylation now remain uncertain. Two studies with mutant mice (Shpargel et al., 2012; Welstead et al., 2012) gave further important insights about the function of both UTY and UTX. The loss of *Utx* was shown to have a profound effect during murine embryogenesis. Interestingly, some X(*Utx*-) Y(+) male mutant mice survived to adulthood, whereas homozygous X(*Utx*-) X(*Utx*-) mutations in females and hemizygous X(*Utx*-) Y(*Uty*-) mutations in males were mid-gestational lethal. This suggests functional redundancy of Utx and Uty. Interestingly, X(*Utx*-) Y(+) male mutant mice were fertile with normal spermatogenesis. Furthermore, in a different study, *Utx* and *Uty* were found to be somewhat differentially expressed in murine brain, likely through differential epigenetic regulation (Xu et al., 2008).

1.5 *ZFX/Y*

ZFX and *ZFY* are a pair of gametologs that encode for Zinc finger proteins (Mardon and Page, 1989; Schneider-Gädicke et al., 1989). Both proteins contain an acidic domain and a potential nucleic acid-binding domain of 13 ”zinc fingers” and likely function as transcription factors (Palmer et al., 1990).

Location, Paralogs and Evolution:
Most species only have one copy of each of the gametologs *ZFX* and *ZFY*. Several species of mice (genus *Mus*), however, have two Y-chromosomal genes (*Zfy-1* and *Zfy-2*) and, additionally, an autosomal copy of the gene (*Zfa*) (Nagamine et al., 1989). *Zfa* is believed to have been retroposed from *Zfx*, has an open reading frame, and is expressed in adult testis, although it remains unclear whether it is functional or not (Ashworth et al., 1990). In knockout mice (*Zfa*-), no abnormal phenotype could be observed (Banks et al., 2003). *ZFX* is located on stratum three according to the older classifications (Lahn and Page, 1999; Ross et al., 2005); (Pandey et al., 2013) place *ZFX* in the seventh of a total of nine strata.

ZFX/ZFY were subject to several studies concerning their evolution and gene conversion. This is a short summary of the findings:

Schneider-Gädicke et al. (1989) first suggested that gene conversion between the human *ZFX* and *ZFY* could offer an explanation for their high similarity, which also extends outside the coding region. Lanfear and Holland (1991) suggest that murine *Zfy-1* and *Zfy-2* sequences, which are markedly divergent members of the *ZFY* gene family, recently underwent rapid sequence divergence, as they are different from all other ZF-sequences. Given that in marsupials and birds both the X- and the Y-linked copies are autosomal

(Bull et al., 1988; Sinclair et al., 1988), it can be assumed that i) the gene duplication leading to the divergence of *ZFX* and *ZFY* predates the mammalian divergence and ii) the genetic isolation of the two genes and their transfer to sex chromosomes predates the divergence of mammals, but occurred after the separation of lineages leading to marsupials and mammals, respectively (Pamilo and Bianchi, 1993; Hayashida et al., 1992). Hayashida et al. (1992) suggest that, in fox and human, gene conversion between the gametologs could explain the unusual divergence pattern found by Lanfear and Holland (1991). They further argue that since human *ZFY* is close to the pseudoautosomal boundary, where X-Y recombination is possible (Schneider-Gädicke et al., 1989), gene conversion might explain the close sequence similarities between parts of human *ZFX* and *ZFY*. In mice, on the other hand, the gene lies near the centromere, making gene conversion more unlikely and resulting in the large evolutionary distance between murine *Zfx* and *Zfy*. Pamilo and Bianchi (1993) compared sequences of *ZFX* (and *Zfa* in case of mice) and *ZFY* (using both *Zfy1* and *Zfy2* in case of mice) of several species (human, mouse, crab-eating fox and chinese hamster plus outgroups). Their findings indicate that the genes have not evolved completely independently since their initial separation, and the authors suggest gene conversion between murine *Zfy-1* and *Zfy-2* and possibly also between gametologs in the crab-eating fox and mice. This is based on the finding that when the 3' and 5' ends of murine and human sequences (*ZFX, Zfx, Zfy-2* and *ZFY*) were aligned separately, conflicting patterns arose: For the 3' half of the gene, gametologs within species showed closer clustering, whereas for the 5' end the opposite was true. According to Pamilo and Bianchi (1993) the trees also suggest that "the X- and Y- chromosomal genes have separated only slightly before the separation of the rodent and primate lineages and that there has been recombination or conversion between the murine genes, homogenizing the 3' ends."

A study by (Drouin et al., 1999) suggests gene conversion events took place between *ZFX* and *ZFY* sometime during the early evolution of primates, on the 3' end of *Zfx/Zfy* of mice and in the middle part of the gene in foxes. For their study, the authors used several different methods, including the co-double method by Balding et al. (1992).

Phylogenetic trees of *ZFX* and *ZFY* have been calculated by Pamilo and Bianchi (1993) and Pecon Slattery et al. (2000). In both cases, unusual clustering of gametologs that was affected by taxonomy could be found (cat *Zfx/Zfy* and fox *Zfx/Zfy* clustered together, respectively). Pecon Slattery et al. (2000) were able to detect gene conversion in the last exon in two different lineages of Felidae by performing a phylogenetic analyses of *Zfx* and *Zfy* among 26 felid species. Interestingly, exclusively gene conversion from the X-chromosome to Y-chromosome was found. The "replacement of the more rapidly evolving Y homolog with the evolutionarily constrained X copy may represent a mechanism for adaptive editing of functional genes on the nonrecombining region of the mammalian Y chromosome" (Pecon Slattery et al., 2000).

Function and Expression:

ZFX controls the self-renewal of human and murine embryonic and adult stem cells (Harel

et al., 2012; Galan-Caridad et al., 2007). As a result of this, mutations in *ZFX* are closely associated with several types of human cancer, and knockdown of *ZFX* by shRNA is considered as a potential therapeutic approach against cancer (see *e.g.,* Fang et al., 2014; Palmer et al., 2014). Murine ZFX has also been shown to play a role in growth and reproductive development (Luoh et al., 1997). Murine *Zfx/Zfy* genes differ from their human homologs in two respects: i) Human *ZFX* escapes X-inactivation, whereas murine *Zfx* does not (Schneider-Gädicke et al., 1989; Adler et al., 1991); and ii) in humans, both *ZFY* and *ZFX* are transcribed ubiquitously (Schneider-Gädicke et al., 1989); this is also the case for murine *Zfx*, but murine *Zfy* is expressed in testes only (Koopman et al., 1991; Mardon et al., 1990).

In mice, *Zfy1, Zfy2* and *Zfx* are expressed during the male-specific interphase between meiosis I and meiosis II and promote the 2nd meiotic division (Vernet et al., 2014). For some time in the past, *ZFY* was in the focus of researchers as a potential sex determining master gene, however, this was soon disproved (Koopman et al., 1989; Sinclair et al., 1988). ZFY potentially plays a role in human and murine spermatogenesis, and ZFY2 removes cells with unpaired chromosomes at the first meiotic metaphase in male mice (Decarpentrie et al., 2012; Vernet et al., 2011).

References

Abid, S., Sagare-Patil, V., Gokral, J., and Modi, D. (2013). Cellular ontogeny of RBMY during human spermatogenesis and its role in sperm motility. Journal of biosciences, **38**(1), 85–92.

Adler, D. A., Bressler, S. L., Chapman, V. M., Page, D. C., and Disteche, C. M. (1991). Inactivation of the Zfx gene on the mouse X chromosome. Proceedings of the National Academy of Sciences of the United States of America, **88**(11), 4592–4595.

Agger, K., Cloos, P. A. C., Christensen, J., Pasini, D., Rose, S., Rappsilber, J., Issaeva, I., Canaani, E., Salcini, A. E., and Helin, K. (2007). UTX and JMJD3 are histone H3K27 demethylases involved in HOX gene regulation and development. Nature, **449**(7163), 731–4.

Ariumi, Y. (2014). Multiple functions of DDX3 RNA helicase in gene regulation, tumorigenesis, and viral infection. Frontiers in Genetics, **5**, 423.

Ashworth, a., Skene, B., Swift, S., and Lovell-Badge, R. (1990). Zfa is an expressed retroposon derived from an alternative transcript of the Zfx gene. The EMBO journal, **9**(5), 1529–34.

Balding, D. J., Nichols, R. A., and Hunt, D. M. (1992). Detecting gene conversion: primate visual pigment genes. Proceedings. Biological sciences / The Royal Society, **249**(1326), 275–80.

Banka, S., Lederer, D., Benoit, V., Jenkins, E., Howard, E., Bunstone, S., Kerr, B., McKee, S., Lloyd, I. C., Shears, D., Stewart, H., White, S. M., Savarirayan, R., Mancini, G. M. S., Beysen, D., Cohn, R. D., Grisart, B., Maystadt, I., and Donnai, D. (2015). Novel KDM6A (UTX) mutations and a clinical and molecular review of the X-linked Kabuki syndrome (KS2). Clinical genetics, **87**(3), 252–8.

Banks, K. G., Johnson, K. A., Lerner, C. P., Mahaffey, C. L., Bronson, R. T., and Simpson, E. M. (2003). Retroposon compensatory mechanism hypothesis not supported: Zfa knockout mice are fertile. Genomics, **82**(3), 254–60.

Bellott, D. W., Hughes, J. F., Skaletsky, H., Brown, L. G., Pyntikova, T., Cho, T.-J., Koutseva, N., Zaghlul, S., Graves, T., Rock, S., Kremitzki, C., Fulton, R. S., Dugan, S., Ding, Y., Morton, D., Khan, Z., Lewis, L., Buhay, C., Wang, Q., Watt, J., Holder, M., Lee, S., Nazareth, L., Rozen, S., Muzny, D. M., Warren, W. C., Gibbs, R. a., Wilson, R. K., and Page, D. C. (2014). Mammalian Y chromosomes retain widely expressed dosage-sensitive regulators. Nature, **508**(7497), 494–9.

Bhowmick, B. K., Satta, Y., and Takahata, N. (2007). The origin and evolution of human ampliconic gene families and ampliconic structure. Genome research, **17**(4), 441–50.

Botlagunta, M., Vesuna, F., Mironchik, Y., Raman, A., Lisok, A., Winnard, P., Mukadam, S., Van Diest, P., Chen, J. H., Farabaugh, P., Patel, A. H., and Raman, V. (2008). Oncogenic role of DDX3 in breast cancer biogenesis. Oncogene, **27**(28), 3912–22.

Brown, G. M., Furlong, R. A., Sargent, C. A., Erickson, R. P., Longepied, G., Mitchell, M., Jones, M. H., Hargreave, T. B., Cooke, H. J., and Affara, N. A. (1998). Characterisation of the coding sequence and fine mapping of the human DFFRY gene and comparative expression analysis and mapping to the Sxrb interval of the mouse Y chromosome of the Dffry gene. Human molecular genetics, **7**(1), 97–107.

Bull, J. J., Hillis, D. M., and O'Steen, S. (1988). Mammalian ZFY sequences exist in reptiles regardless of sex-determining mechanism. Science (New York, N.Y.), **242**(4878), 567–9.

Chai, N. N., Zhou, H., Hernandez, J., Najmabadi, H., Bhasin, S., and Yen, P. H. (1998). Structure and organization of the RBMY genes on the human Y chromosome: transposition and amplification of an ancestral autosomal hnRNPG gene. Genomics, **49**(2), 283–9.

Chang, T.-C. and Liu, W.-S. (2010). The molecular evolution of PL10 homologs. BMC evolutionary biology, **10**(1), 127.

Chao, C.-H., Chen, C.-M., Cheng, P.-L., Shih, J.-W., Tsou, A.-P., and Lee, Y.-H. W. (2006). DDX3, a DEAD box RNA helicase with tumor growth-suppressive property and transcriptional regulation activity of the p21waf1/cip1 promoter, is a candidate tumor suppressor. Cancer research, **66**(13), 6579–88.

Cho, Y.-W., Hong, T., Hong, S., Guo, H., Yu, H., Kim, D., Guszczynski, T., Dressler, G. R., Copeland, T. D., Kalkum, M., and Ge, K. (2007). PTIP associates with MLL3- and MLL4-containing histone H3 lysine 4 methyltransferase complex. The Journal of biological chemistry, **282**(28), 20395–406.

Decarpentrie, F., Vernet, N., Mahadevaiah, S. K., Longepied, G., Streichemberger, E., Aknin-Seifer, I., Ojarikre, O. A., Burgoyne, P. S., Metzler-Guillemain, C., and Mitchell, M. J. (2012). Human and mouse ZFY genes produce a conserved testis-specific transcript encoding a zinc finger protein with a short acidic domain and modified transactivation potential. Human molecular genetics, **21**(12), 2631–45.

Delbridge, M. L., Lingenfelter, P. A., Disteche, C. M., and Graves, J. A. (1999). The candidate spermatogenesis gene RBMY has a homologue on the human X chromosome. Nature genetics, **22**(3), 223–4.

Ditton, H. J., Zimmer, J., Kamp, C., Rajpert-De Meyts, E., and Vogt, P. H. (2004). The AZFa gene DBY (DDX3Y) is widely transcribed but the protein is limited to the male germ cells by translation control. Human molecular genetics, **13**(19), 2333–41.

Drouin, G., Prat, F., Ell, M., and Clarke, G. D. (1999). Detecting and characterizing gene conversions between multigene family members. Molecular biology and evolution, **16**(Hughes), 1369–1390.

Elliott, D. J., Millar, M. R., Oghene, K., Ross, A., Kiesewetter, F., Pryor, J., McIntyre, M., Hargreave, T. B., Saunders, P. T., Vogt, P. H., Chandley, A. C., and Cooke, H. (1997). Expression of RBM in the nuclei of human germ cells is dependent on a critical region of the Y chromosome long arm. Proceedings of the National Academy of Sciences of the United States of America, **94**(8), 3848–53.

Elliott, D. J., Venables, J. P., Newton, C. S., Lawson, D., Boyle, S., Eperon, I. C., and Cooke, H. J. (2000). An evolutionarily conserved germ cell-specific hnRNP is encoded by a retrotransposed gene. Human molecular genetics, **9**(14), 2117–2124.

Fang, Q., Fu, W.-H., Yang, J., Li, X., Zhou, Z.-S., Chen, Z.-W., and Pan, J.-H. (2014). Knockdown of ZFX suppresses renal carcinoma cell growth and induces apoptosis. Cancer genetics.

Foresta, C., Moro, E., Rossi, A., Rossato, M., Garolla, A., and Ferlin, A. (2000). Role of the AZFa candidate genes in male infertility. Journal of endocrinological investigation, **23**(10), 646–51.

Franca, R., Belfiore, A., Spadari, S., and Maga, G. (2007). Human DEAD-box ATPase DDX3 shows a relaxed nucleoside substrate specificity. Proteins, **67**(4), 1128–37.

Galan-Caridad, J. M., Harel, S., Arenzana, T. L., Hou, Z. E., Doetsch, F. K., Mirny, L. A., and Reizis, B. (2007). Zfx controls the self-renewal of embryonic and hematopoietic stem cells. Cell, **129**(2), 345–57.

Gale, M. and Foy, E. M. (2005). Evasion of intracellular host defence by hepatitis C virus. Nature, **436**(7053), 939–45.

Gerrard, D. T. and Filatov, D. A. (2005). Positive and negative selection on mammalian Y chromosomes. Molecular biology and evolution, **22**(6), 1423–32.

Greenfield, A., Scott, D., Pennisi, D., Ehrmann, I., Ellis, P., Cooper, L., Simpson, E., and Koopman, P. (1996). An H-YDb epitope is encoded by a novel mouse Y chromosome gene. Nature genetics, **14**(4), 474–8.

Greenfield, A., Carrel, L., Pennisi, D., Philippe, C., Quaderi, N., Siggers, P., Steiner, K., Tam, P. P., Monaco, A. P., Willard, H. F., and Koopman, P. (1998). The UTX gene escapes X inactivation in mice and humans. Human molecular genetics, **7**(4), 737–42.

Hall, N. M., Brown, G. M., Furlong, R. A., Sargent, C. A., Mitchell, M., Rocha, D., and Affara, N. A. (2003). Usp9y (ubiquitin-specific protease 9 gene on the Y) is associated

with a functional promoter and encodes an intact open reading frame homologous to Usp9x that is under selective constraint. Mammalian genome : official journal of the International Mammalian Genome Society, **14**(7), 437–47.

Harel, S., Tu, E. Y., Weisberg, S., Esquilin, M., Chambers, S. M., Liu, B., Carson, C. T., Studer, L., Reizis, B., and Tomishima, M. J. (2012). ZFX Controls the Self-Renewal of Human Embryonic Stem Cells. PLoS ONE, **7**(8), e42302.

Hayashida, H., Kuma, K., and Miyata, T. (1992). Interchromosomal Gene Conversion as a Possible Mechanism for Explaining Divergence Patterns of Zfy-Related Genes. Journal of Molecular Evolution, **35**(2), 181–183.

Heinrich, B., Zhang, Z., Raitskin, O., Hiller, M., Benderska, N., Hartmann, A. M., Bracco, L., Elliott, D., Ben-Ari, S., Soreq, H., Sperling, J., Sperling, R., and Stamm, S. (2009). Heterogeneous nuclear ribonucleoprotein G regulates splice site selection by binding to CC(A/C)-rich regions in pre-mRNA. The Journal of biological chemistry, **284**(21), 14303–15.

Homan, C. C., Kumar, R., Nguyen, L. S., Haan, E., Raymond, F. L., Abidi, F., Raynaud, M., Schwartz, C. E., Wood, S. A., Gecz, J., and Jolly, L. A. (2014). Mutations in USP9X are associated with X-linked intellectual disability and disrupt neuronal cell migration and growth. American journal of human genetics, **94**(3), 470–8.

Hong, S., Cho, Y.-W., Yu, L.-R., Yu, H., Veenstra, T. D., and Ge, K. (2007). Identification of JmjC domain-containing UTX and JMJD3 as histone H3 lysine 27 demethylases. Proceedings of the National Academy of Sciences of the United States of America, **104**(47), 18439–44.

Jones, M. H., Furlong, R. A., Burkin, H., Chalmers, I. J., Brown, G. M., Khwaja, O., and Affara, N. A. (1996). The Drosophila developmental gene fat facets has a human homologue in Xp11.4 which escapes X-inactivation and has related sequences on Yq11.2. Human Molecular Genetics, **5**(11), 1695–1701.

Karro, J. E., Yan, Y., Zheng, D., Zhang, Z., Carriero, N., Cayting, P., Harrrison, P., and Gerstein, M. (2007). Pseudogene.org: a comprehensive database and comparison platform for pseudogene annotation. Nucleic acids research, **35**(Database issue), D55–60.

Khut, P.-Y., Tucker, B., Lardelli, M., and Wood, S. a. (2007). Evolutionary and expression analysis of the zebrafish deubiquitylating enzyme, usp9. Zebrafish, **4**(2), 95–101.

Kim, Y.-S., Lee, S.-G., Park, S. H., and Song, K. (2001). Gene Structure of the Human DDX3 and Chromosome Mapping of Its Related Sequences. Molecules and Cells, **12**(2), 209–214.

Koopman, P., Gubbay, J., Collignon, J., and Lovell-Badge, R. (1989). Zfy gene expression patterns are not compatible with a primary role in mouse sex determination. Nature, **342**(6252), 940–2.

Koopman, P., Ashworth, A., and Lovell-Badge, R. (1991). The ZFY gene family in humans and mice. Trends in genetics : TIG, **7**(4), 132–136.

Krausz, C., Degl'Innocenti, S., Nuti, F., Morelli, A., Felici, F., Sansone, M., Varriale, G., and Forti, G. (2006). Natural transmission of USP9Y gene mutations: a new perspective on the role of AZFa genes in male fertility. Human molecular genetics, **15**(18), 2673–81.

Laaser, I., Theis, F. J., de Angelis, M. H., Kolb, H.-J., and Adamski, J. (2011). Huge splicing frequency in human Y chromosomal UTY gene. Omics : a journal of integrative biology, **15**(3), 141–54.

Lahn, B. and Page, D. (1997). Functional coherence of the human Y chromosome. Science.

Lahn, B. and Page, D. (1999). Four evolutionary strata on the human X chromosome. Science, **286**, 964–967.

Lai, M.-C., Chang, W.-C., Shieh, S.-Y., and Tarn, W.-Y. (2010). DDX3 regulates cell growth through translational control of cyclin E1. Molecular and cellular biology, **30**(22), 5444–53.

Lanfear, J. and Holland, P. W. H. (1991). The molecular evolution of ZFY-related genes in birds and mammals. Journal of Molecular Evolution, **32**(4), 310–315.

Lee, C.-S., Dias, A. P., Jedrychowski, M., Patel, A. H., Hsu, J. L., and Reed, R. (2008). Human DDX3 functions in translation and interacts with the translation initiation factor eIF3. Nucleic acids research, **36**(14), 4708–18.

Lee, K. H., Song, G. J., Kang, I. S., Kim, S. W., Paick, J.-S., Chung, C. H., and Rhee, K. (2003). Ubiquitin-specific protease activity of USP9Y, a male infertility gene on the Y chromosome. Reproduction, fertility, and development, **15**(1-2), 129–33.

Lingenfelter, P. A., Delbridge, M. L., Thomas, S., Hoekstra, H. E., Mitchell, M. J., Graves, J. A., and Disteche, C. M. (2001). Expression and conservation of processed copies of the RBMX gene. Mammalian genome : official journal of the International Mammalian Genome Society, **12**(7), 538–45.

Liu, J., Mercher, T., Scholl, C., Brumme, K., Gilliland, D. G., and Zhu, N. (2012). A functional role for the histone demethylase UTX in normal and malignant hematopoietic cells. Experimental hematology, **40**(6), 487–98.e3.

Liu, W.-S., Wang, A., Yang, Y., Chang, T.-C., Landrito, E., and Yasue, H. (2009). Molecular Characterization of the DDX3Y Gene and Its Homologs in Cattle. Cytogenetic and Genome Research, **126**(4), 318–328.

Luddi, A., Margollicci, M., Gambera, L., Serafini, F., Cioni, M., De Leo, V., Balestri, P., and Piomboni, P. (2009). Spermatogenesis in a man with complete deletion of USP9Y. The New England journal of medicine, **360**(9), 881–5.

Luoh, S. W., Bain, P. a., Polakiewicz, R. D., Goodheart, M. L., Gardner, H., Jaenisch, R., and Page, D. C. (1997). Zfx mutation results in small animal size and reduced germ cell number in male and female mice. Development (Cambridge, England), **124**, 2275–2284.

Mansour, A. A., Gafni, O., Weinberger, L., Zviran, A., Ayyash, M., Rais, Y., Krupalnik, V., Zerbib, M., Amann-Zalcenstein, D., Maza, I., Geula, S., Viukov, S., Holtzman, L., Pribluda, A., Canaani, E., Horn-Saban, S., Amit, I., Novershtern, N., and Hanna, J. H. (2012). The H3K27 demethylase Utx regulates somatic and germ cell epigenetic reprogramming. Nature, **488**(7411), 409–13.

Mardon, G. and Page, D. C. (1989). The sex-determining region of the mouse Y chromosome encodes a protein with a highly acidic domain and 13 zinc fingers. Cell, **56**(5), 765–770.

Mardon, G., Luoh, S. W., Simpson, E. M., Gill, G., Brown, L. G., and Page, D. C. (1990). Mouse Zfx protein is similar to Zfy-2: each contains an acidic activating domain and 13 zinc fingers. Molecular and cellular biology, **10**(2), 681–8.

Matsunaga, S., Takata, H., Morimoto, A., Hayashihara, K., Higashi, T., Akatsuchi, K., Mizusawa, E., Yamakawa, M., Ashida, M., Matsunaga, T. M., Azuma, T., Uchiyama, S., and Fukui, K. (2012). RBMX: a regulator for maintenance and centromeric protection of sister chromatid cohesion. Cell reports, **1**(4), 299–308.

Mazeyrat, S., Saut, N., Sargent, C. a., Grimmond, S., Longepied, G., Ehrmann, I. E., Ellis, P. S., Greenfield, A., Affara, N. a., and Mitchell, M. J. (1998). The mouse Y chromosome interval necessary for spermatogonial proliferation is gene dense with syntenic homology to the human AZFa region. Human Molecular Genetics, **7**(11), 1713–1724.

Mazeyrat, S., Saut, N., Mattei, M. G., and Mitchell, M. J. (1999). RBMY evolved on the Y chromosome from a ubiquitously transcribed X-Y identical gene. Nature genetics, **22**(3), 224–6.

McKee, A. E., Minet, E., Stern, C., Riahi, S., Stiles, C. D., and Silver, P. A. (2005). A genome-wide in situ hybridization map of RNA-binding proteins reveals anatomically restricted expression in the developing mouse brain. BMC developmental biology, **5**(1), 14.

Miller, S. a., Mohn, S. E., and Weinmann, A. S. (2010). Jmjd3 and UTX play a demethylase-independent role in chromatin remodeling to regulate t-box family member-dependent gene expression. Molecular Cell, **40**(4), 594–605.

Morales Torres, C., Laugesen, A., and Helin, K. (2013). Utx is required for proper induction of ectoderm and mesoderm during differentiation of embryonic stem cells. PloS one, **8**(4), e60020.

Murtaza, M., Jolly, L. A., Gecz, J., and Wood, S. A. (2015). La FAM fatale: USP9X in development and disease. Cellular and molecular life sciences : CMLS.

Nagamine, C. M., Chan, K. M., Kozak, C. a., and Lau, Y. F. (1989). Chromosome mapping and expression of a putative testis-determining gene in mouse. Science (New York, N.Y.), **243**(4887), 80–3.

Owsianka, A. (1999). Hepatitis C Virus Core Protein Interacts with a Human DEAD Box Protein DDX3. Virology, **257**(2), 330–340.

Palmer, C. J., Galan-Caridad, J. M., Weisberg, S. P., Lei, L., Esquilin, J. M., Croft, G. F., Wainwright, B., Canoll, P., Owens, D. M., and Reizis, B. (2014). Zfx facilitates tumorigenesis caused by activation of the Hedgehog pathway. Cancer research, **74**(20), 5914–24.

Palmer, M. S., Berta, P., Sinclair, a. H., Pym, B., and Goodfellow, P. N. (1990). Comparison of human zfy and zfx transcripts. In Proceedings of the National Academy of Sciences of the United States of America, volume 87, pages 1681–1685.

Pamilo, P. and Bianchi, N. O. (1993). Evolution of the Zfx and Zfy genes: rates and interdependence between the genes. Molecular Biology and Evolution, **10**(2), 271–281.

Pandey, R. S., Wilson Sayres, M. A., and Azad, R. K. (2013). Detecting evolutionary strata on the human x chromosome in the absence of gametologous y-linked sequences. Genome biology and evolution, **5**(10), 1863–71.

Park, S. H., Lee, S. G., Kim, Y., and Song, K. (1998). Assignment of a human putative RNA helicase gene, DDX3, to human X chromosome bands p11.3–¿p11.23. Cytogenetics and cell genetics, **81**(3-4), 178–9.

Pecon Slattery, J., Sanner-Wachter, L., and O'Brien, S. J. (2000). Novel gene conversion between X-Y homologues located in the nonrecombining region of the Y chromosome in Felidae (Mammalia). Proceedings of the National Academy of Sciences of the United States of America, **97**(10), 5307–12.

Rosner, a. and Rinkevich, B. (2007). The DDX3 subfamily of the DEAD box helicases: divergent roles as unveiled by studying different organisms and in vitro assays. Current medicinal chemistry, **14**(23), 2517–2525.

Ross, M., Grafham, D. V., Coffey, A., and et al. (2005). The DNA sequence of the human X chromosome. Nature, **434**, 325–337.

Saito, T. and Gale, M. (2007). Principles of intracellular viral recognition. Current Opinion in Immunology, **19**(1), 17–23.

Schneider-Gädicke, a., Beer-Romero, P., Brown, L. G., Nussbaum, R., and Page, D. C. (1989). ZFX has a gene structure similar to ZFY, the putative human sex determinant, and escapes X inactivation. Cell, **57**(7), 1247–58.

Sekiguchi, T., Iida, H., Fukumura, J., and Nishimoto, T. (2004). Human DDX3Y, the Y-encoded isoform of RNA helicase DDX3, rescues a hamster temperature-sensitive ET24 mutant cell line with a DDX3X mutation. Experimental cell research, **300**(1), 213–22.

Session, D. R., Lee, G. S., and Wolgemuth, D. J. (2001). Characterization of D1Pas1, a mouse autosomal homologue of the human AZFa region DBY, as a nuclear protein in spermatogenic cells. Fertility and sterility, **76**(4), 804–11.

Shahhoseini, M., Taghizadeh, Z., Hatami, M., and Baharvand, H. (2013). Retinoic acid dependent histone 3 demethylation of the clustered HOX genes during neural differentiation of human embryonic stem cells. Biochemistry and cell biology = Biochimie et biologie cellulaire, **91**(2), 116–22.

Shpargel, K. B., Sengoku, T., Yokoyama, S., and Magnuson, T. (2012). UTX and UTY demonstrate histone demethylase-independent function in mouse embryonic development. PLoS genetics, **8**(9), e1002964.

Sinclair, A. H., Foster, J. W., Spencer, J. A., Page, D. C., Palmer, M., Goodfellow, P. N., and Graves, J. A. (1988). Sequences homologous to ZFY, a candidate human sex-determining gene, are autosomal in marsupials. Nature, **336**(6201), 780–3.

Stegeman, S., Jolly, L. A., Premarathne, S., Gecz, J., Richards, L. J., Mackay-Sim, A., and Wood, S. A. (2013). Loss of Usp9x disrupts cortical architecture, hippocampal development and TGFβ-mediated axonogenesis. PloS one, **8**(7), e68287.

Sun, M., Song, L., Li, Y., Zhou, T., and Jope, R. S. (2008). Identification of an anti-apoptotic protein complex at death receptors. Cell death and differentiation, **15**(12), 1887–900.

Tarpey, P. S., Smith, R., Pleasance, E., Whibley, A., Edkins, S., Hardy, C., O'Meara, S., Latimer, C., Dicks, E., Menzies, A., Stephens, P., Blow, M., Greenman, C., Xue, Y., Tyler-Smith, C., Thompson, D., Gray, K., Andrews, J., Barthorpe, S., Buck, G., Cole, J., Dunmore, R., Jones, D., Maddison, M., Mironenko, T., Turner, R., Turrell, K., Varian, J., West, S., Widaa, S., Wray, P., Teague, J., Butler, A., Jenkinson, A., Jia, M., Richardson, D., Shepherd, R., Wooster, R., Tejada, M. I., Martinez, F., Carvill, G., Goliath, R., de Brouwer, A. P. M., van Bokhoven, H., Van Esch, H., Chelly, J., Raynaud, M., Ropers, H.-H., Abidi, F. E., Srivastava, A. K., Cox, J., Luo, Y., Mallya,

U., Moon, J., Parnau, J., Mohammed, S., Tolmie, J. L., Shoubridge, C., Corbett, M., Gardner, A., Haan, E., Rujirabanjerd, S., Shaw, M., Vandeleur, L., Fullston, T., Easton, D. F., Boyle, J., Partington, M., Hackett, A., Field, M., Skinner, C., Stevenson, R. E., Bobrow, M., Turner, G., Schwartz, C. E., Gecz, J., Raymond, F. L., Futreal, P. A., and Stratton, M. R. (2009). A systematic, large-scale resequencing screen of X-chromosome coding exons in mental retardation. Nature genetics, **41**(5), 535–43.

Tsend-Ayush, E., O'Sullivan, L. A., Grützner, F. S., Onnebo, S. M. N., Lewis, R. S., Delbridge, M. L., Marshall Graves, J. A., and Ward, A. C. (2005). RBMX gene is essential for brain development in zebrafish. Developmental dynamics : an official publication of the American Association of Anatomists, **234**(3), 682–8.

Tsuei, D.-J., Hsu, H.-C., Lee, P.-H., Jeng, Y.-M., Pu, Y.-S., Chen, C.-N., Lee, Y.-C., Chou, W.-C., Chang, C.-J., Ni, Y.-H., and Chang, M.-H. (2004). RBMY, a male germ cell-specific RNA-binding protein, activated in human liver cancers and transforms rodent fibroblasts. Oncogene, **23**(34), 5815–22.

van Haaften, G., Dalgliesh, G. L., Davies, H., Chen, L., Bignell, G., Greenman, C., Edkins, S., Hardy, C., O'Meara, S., Teague, J., Butler, A., Hinton, J., Latimer, C., Andrews, J., Barthorpe, S., Beare, D., Buck, G., Campbell, P. J., Cole, J., Forbes, S., Jia, M., Jones, D., Kok, C. Y., Leroy, C., Lin, M.-L., McBride, D. J., Maddison, M., Maquire, S., McLay, K., Menzies, A., Mironenko, T., Mulderrig, L., Mudie, L., Pleasance, E., Shepherd, R., Smith, R., Stebbings, L., Stephens, P., Tang, G., Tarpey, P. S., Turner, R., Turrell, K., Varian, J., West, S., Widaa, S., Wray, P., Collins, V. P., Ichimura, K., Law, S., Wong, J., Yuen, S. T., Leung, S. Y., Tonon, G., DePinho, R. A., Tai, Y.-T., Anderson, K. C., Kahnoski, R. J., Massie, A., Khoo, S. K., Teh, B. T., Stratton, M. R., and Futreal, P. A. (2009). Somatic mutations of the histone H3K27 demethylase gene UTX in human cancer. Nature genetics, **41**(5), 521–3.

Vernet, N., Mahadevaiah, S. K., Ojarikre, O. A., Longepied, G., Prosser, H. M., Bradley, A., Mitchell, M. J., and Burgoyne, P. S. (2011). The Y-encoded gene zfy2 acts to remove cells with unpaired chromosomes at the first meiotic metaphase in male mice. Current biology : CB, **21**(9), 787–93.

Vernet, N., Mahadevaiah, S. K., Yamauchi, Y., Decarpentrie, F., Mitchell, M. J., Ward, M. A., and Burgoyne, P. S. (2014). Mouse Y-linked Zfy1 and Zfy2 are expressed during the male-specific interphase between meiosis I and meiosis II and promote the 2nd meiotic division. PLoS genetics, **10**(6), e1004444.

Vogt, P. H., Edelmann, A., Kirsch, S., Henegariu, O., Hirschmann, P., Kiesewetter, F., Köhn, F. M., Schill, W. B., Farah, S., Ramos, C., Hartmann, M., Hartschuh, W., Meschede, D., Behre, H. M., Castel, A., Nieschlag, E., Weidner, W., Gröne, H. J., Jung, A., Engel, W., and Haidl, G. (1996). Human Y chromosome azoospermia factors

(AZF) mapped to different subregions in Yq11. Human molecular genetics, **5**(7), 933–43.

Vong, Q. P., Li, Y., Lau, Y.-F. C., Dym, M., Rennert, O. M., and Chan, W.-Y. (2006). Structural characterization and expression studies of Dby and its homologs in the mouse. Journal of andrology, **27**(5), 653–61.

Walport, L. J., Hopkinson, R. J., Vollmar, M., Madden, S. K., Gileadi, C., Oppermann, U., Schofield, C. J., and Johansson, C. (2014). Human UTY(KDM6C) is a male-specific N-methyl lysyl demethylase. The Journal of biological chemistry, **289**(26), 18302–13.

Wang, C., Lee, J.-E., Cho, Y.-W., Xiao, Y., Jin, Q., Liu, C., and Ge, K. (2012). UTX regulates mesoderm differentiation of embryonic stem cells independent of H3K27 demethylase activity. Proceedings of the National Academy of Sciences of the United States of America, **109**(38), 15324–9.

Warren, E. H., Gavin, M. a., Simpson, E., Chandler, P., Page, D. C., Disteche, C., Stankey, K. a., Greenberg, P. D., and Riddell, S. R. (2000). The human UTY gene encodes a novel HLA-B8-restricted H-Y antigen. Journal of immunology (Baltimore, Md. : 1950), **164**(5), 2807–2814.

Welstead, G. G., Creyghton, M. P., Bilodeau, S., Cheng, A. W., Markoulaki, S., Young, R. A., and Jaenisch, R. (2012). X-linked H3K27me3 demethylase Utx is required for embryonic development in a sex-specific manner. Proceedings of the National Academy of Sciences of the United States of America, **109**(32), 13004–9.

Wyckoff, G. J., Li, J., and Wu, C.-I. (2002). Molecular Evolution of Functional Genes on the Mammalian Y Chromosome. Molecular Biology and Evolution, **19**(9), 1633–1636.

Xu, J., Burgoyne, P. S., and Arnold, A. P. (2002). Sex differences in sex chromosome gene expression in mouse brain. Human molecular genetics, **11**(12), 1409–1419.

Xu, J., Deng, X., Watkins, R., and Disteche, C. M. (2008). Sex-specific differences in expression of histone demethylases Utx and Uty in mouse brain and neurons. The Journal of neuroscience : the official journal of the Society for Neuroscience, **28**(17), 4521–7.

Yang, F., Babak, T., Shendure, J., and Disteche, C. M. (2010). Global survey of escape from X inactivation by RNA-sequencing in mouse. Genome research, **20**(5), 614–22.

Yedavalli, V. S. R. K., Neuveut, C., Chi, Y.-H., Kleiman, L., and Jeang, K.-T. (2004). Requirement of DDX3 DEAD box RNA helicase for HIV-1 Rev-RRE export function. Cell, **119**(3), 381–92.
